# Preclinical Evaluation of ^68^Ga-MAA from Commercial Available ^99m^Tc-MAA Kit

**Published:** 2017

**Authors:** Saeed Shanehsazzadeh, Amir Reza Jalilian, Afsaneh Lahooti, Parham Geramifar, Davood Beiki, Hassan Yousefnia, Amir Rabiee, Mohammad Mazidi, Seyedeh Fatemeh Mirshojaei, Stephan Maus

**Affiliations:** a *Radiation Application Research School, Nuclear Science and Technology Research Institute (NSTRI), Tehran, Iran. *; b *Department of Medical Physics and Biomedical Engineering, Faculty of Medicine, Tehran University of Medical Science, Iran. *; c *Research Center for Nuclear Medicine, Shariati Hospital, Tehran University of Medical Sciences, Tehran, Iran. *; d *Clinic of Nuclear Medicine, University Medical Centre Mainz, Langenbeckstrasse 1, D-55131 Mainz, Germany.*

**Keywords:** Biodistribution, Macroaggregated Albumin, ^68^Ga, Imaging, pulmonary ventilation/perfusion scan

## Abstract

^99m^Tc-Macroaggregated Albumin (^99m^Tc-MAA) has been used as a perfusion agent. This study described development of the ^68^Ga-MAA via commercially available kits from Pars-Isotopes Company as a ^99m^Tc-MAA kit. ^68^Ge/^68^Ga generator was eluted with suprapure HCl (0.6 M, 6 mL) in 0.5 mL fractions. The two fractions with the highest ^68^GaCl_3_ activity were generally used for labeling purposes. After labeling, the final product was centrifuged 2 times to purify the solution. Five rats were sacrificed at each exact time interval (from 15 min to 2 h post injection) and the percentage of injected dose per gram (%ID/g) of each organ was measured by direct counting from 11 harvested organs of rats. The RTLC showed that labeling yields before centrifuges were 90% and 95% for Pars-Isotopes and GE kits, respectively and after centrifuges, they became 100%. The microscopic size examination showed a shift in the particle sizes post centrifuges and the biodistribution data revealed the efficiency and benefits of centrifuges in terms of preventing the of liver and bone marrow uptakes especially for Pars-Isotopes kits. Our results showed that after centrifuges of the final product, the lung uptakes increased from 89% to more than 97% of %ID/g after 5 min post injections. The whole procedure took less than 25 min from elution to the ﬁnal product. Since ^99m^Tc-MAA remained longer than ^68^Ga-MAA in the lung and ^68^Ga-MAA showed better image qualities, using ^68^Ga-MAA is recommended.

## Introduction

A pulmonary ventilation/perfusion scan (V/Q scan) involves two nuclear scan tests to measure breathing (ventilation) and circulation (perfusion) in all areas of the lungs. Nowadays, during the perfusion scan, the intravenous injection (i.v.) of radioactive technetium macro aggregated albumin (^99m^Tc-MAA). A gamma camera acquires the images for both phases of the study. A SPECT image can also be taken following an injection of commercially used particles are MAA, which are labeled with ^99m^Tc (1, 2). These micro particles are 15–100 μm in size and lodge in the pulmonary capillaries and in the pre-capillary arterioles. The particle distribution accurately defines regional lung perfusion. When performing the study, an important factor is the number of particles injected and the size distribution of the particles (1). A minimum of 60,000 particles are required to obtain uniform distribution of activity reflecting regional perfusion (3). Normally, about 400,000 labeled particles are injected. Bearing in mind that there are over 280 billion pulmonary capillaries and 300 million pre-capillary arterioles, the administration of up to 400,000 particles will result in obstruction of only a very small fraction of pulmonary vessels. A reduction in the number of particles administered to between 100,000 and 200,000 is recommended in patients with known pulmonary hypertension, the right to left heart shunt or after single lung transplantation. In infants and children, the number of particles may be further reduced in accordance with patient’s weight (4). 

Due to some physical limitations for SPECT such as spatial resolution, it would be better to perform the scan with positron emission tomography (PET) rather than conventional gamma camera scintigraphy (5, 6). This has been performed with Gallium-68 labeled carbon nanoparticles (Galligas) using a conventional Technegas machine for ventilation images, and with Gallium-68 labeled MAA (^68^Ga-MAA) for perfusion images. PET has multiple potential benefits including superior resolution, speed and quantification (5).

^68^Ga is an excellent positron emitting radioisotope suitable for clinical PET scan applications (7). The physical characteristics of this radioisotope (positron emission (89%), low abundance of 1,077 keV photon emission (3.22%) and the relatively short half-life (*t½ *= 67.71 min) (8). permit PET applications of the ^68^Ga-radiopharmacuticals, while maintaining an acceptable radiation dose to the patient. Besides to these specifications, the cyclotron independent availability of ^68^Ga from a ^68^Ge/^68^Ga generator at a reasonable cost makes it an attractive and realistic option for countries with limited or no cyclotron facilities.

Although there are several studies that compare the results of ^99m^Tc-MAA with ^68^Ga-MAA but there is still lack of data about the distribution of ^68^Ga-MAA in the body especially in the first minutes post injections and the clearance of ^68^Ga-MAA from the blood (9-11). 

In continues to our previous studies and with the aim of developing new diagnostic agents in our country (12-14), in this study, we tried to develop and optimize the production of ^68^Ga-MAA via commercially available kits from GE and Pars-Isotopes kits for ^99m^Tc-MAA kits.

## Experimental

^68^Ge/^68^Ga generator was eluted with suprapure HCl (0.6 M, 6 mL) in 0.5 mL fractions. The two fractions with the highest ^68^GaCl_3_ (700-900 MBq) activity were generally used for labeling purposes. The MAA kits were obtained from Pars-Isotope Company (TCK-Pars-1800) which contain 3mg HAS (human serum albumin). All other chemical reagents were purchased from Sigma-Aldrich Chemical Co. U.K. Whatman paper No. 2 was obtained from Whatman (UK). Radio-chromatography was performed by Whatman paper using a thin layer chromatography scanner, Bioscan AR 2000, Paris, France. The activity of the samples were measured by a p-type coaxial high-purity germanium (HPGe) detector (model: EGPC 80-200R) coupled with a multichannel analyzer system. Calculations were based on the 511 keV peak for ^68^Ga. All values were expressed as mean ± standard deviation (Mean ± SD). Animal studies were performed in accordance with the United Kingdom biological council´s guidelines on the use of living animals in scientific investigations, second edition (15). 

*Size measurements*

The particle size distributions were measured via sedimentation in Andreasen cylinders and via laser diffraction (using the Fraunhofer approximation) on a Fritsch Particle Sizer Analysette 22 Nanotec (Fritsch Laborgerätebau GmbH, Germany). The size of the particles was measured pre and post labeling.

*Preparation of MAA from MAA kits*

The general procedure for the preparation of ^99m^Tc labeled commercial human serum MAA kits was based on and similar to the process described by previous studies (11, 16, 17). Each kit was suspended in 10 mL of 0.9% sodium chloride and vigorously mixed for 15 min to make the particles free of additional compounds such as stannous chloride dihydrates. After centrifuges (at 4000 rpm for 5 min), the supernatants were wasted and the retained particles were reconstituted in 0.3–0.5 mL sterile water and ready for radioactive labeling.

*Labeling of MAA with* ^68^ *Ga*

The MAA particles were mixed with 1.5 mL of ^68^Ge/^68^Ga generator eluted (contains 600MBq) and the pH adjusted to 4 with 500 mg of HEPES buffer. The mixture was allowed to react and stirred gently for 8 min at 75 °C. The labeled compound then quenched via 10 mL of sterile water. In order to purify the ^68^Ga-MAA and get rid of free ^68^Ga the sample were centrifuged again for 5 min (at 4000 rpm) and supernatant were squandered. 

*Determination of labeling yields*

**Figure 1.**
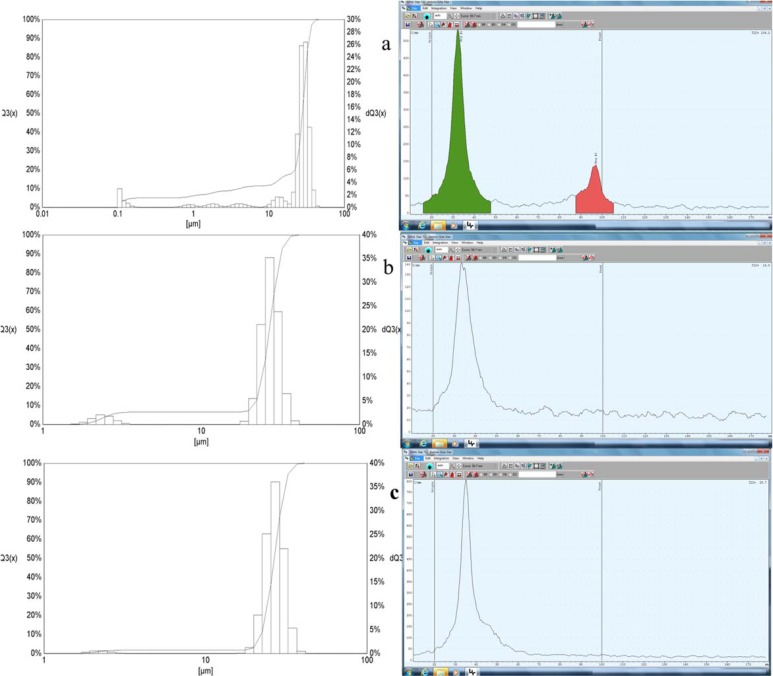
The RTLC results (on the right sides) and the Fritsch Particle sizer results (left side) of the MAA a) after labeling with ^68^Ga b) after first centrifuge and c) after 2 times centrifuge post labeling. The X and Y axises for the Fritsch Particle sizer were the mean size diameter and the intensity, respectively

In order to determine the labeling yields, we used different methods of radio thin layer chromatography (RTLC) to determine the radioactive labeling yields. We used RTLC on silica gel impregnated glass fiber sheets as the stationary phase and 0.9% NaCl solution as the mobile phase. The ^68^Ga labeled with MAA remained at the start position and the free ^68^Ga migrated with the solvent front. The percentages of each fraction were determined relative to the total activity of the chromatogram (Figure 1). As represented in Figure 1, the labeling yield was about 95% therefore we considered to centrifuge the compound again for 5 min (at 4000 rpm). The RTLC results showed that there is no free activity.

*Animal Models*

For biodistribution evaluation of the ^68^Ga-MAA, normal female wild-type rats were used (purchased from Razi Institute, Karaj, Iran). During the entire study, autoclaved food and drinking water were available *ad libitum*

*Biodistribution and Imaging in wild-type rats*

The distribution of radiolabeled complex (15, 30, 45, 60 and 120 min) among tissues was determined in wild-type rats. The total amount of radioactivity injected into each rat was measured by counting the syringe before and after injection in a dose calibrator with fixed geometry. The rats were sacrificed using the animal care protocols at selected times after injection. Blood samples were rapidly taken from the rats’ aorta after scarification. The tissues (heart, lung, intestine, skin, stomach, kidneys, spleen, liver, muscle, urine, carcass and bone) were weighed and rinsed with normal saline and their specific activities were determined with an HPGe detector (count 3 times) and the percentage of injected dose per gram (%ID/g) of each organ was measured (18, 19).

*Measurement of activity*

The activity in the syringes was measured before and after administration of the radiopharmaceutical with well-type ionization chamber (CRC-15R, Capintec, USA N.J.). All samples were background subtracted, the decay correction was not performed and then similar samples were averaged together (20).

For each of these measurements, three samples (from each organ) were weighed and then counted by HPGe to determine the percentage of injected dose per gram (which was equivalent to the percentage of injected activity per gram %IA/g≡%ID/g); all the organ activity measurements were normalized to injected activity. Uncertainties in the determinations were minimal because each assay collected at least 10,000 counts, which results the standard deviation (SD) of less than 1%. In all the measurements, we tried to keep same geometry and volume in order to prevent overestimation and underestimation in dose measurements. All samples became background subtracted and decay correction was not considered for all measurements and the similar samples were averaged together (18, 21).

The HPGe detector gave us the counts, therefore we used the below formula to convert the counts into the activity (22): 

Equ 1 $${A(\mathrm{Bq})}_{\mathrm{Tissue}}=\frac{\mathrm{Area}}{\mathrm{t*Eff*Br}}$$

Where *t* is the time of counts and *Eff* is the efficiency of the detector for the selected energy and *Br* is the decay yield of selected energy (512 keV) for the ^68^Ga.

The ^68^Ga activity concentration at time t, %ID/g (t) was then calculated as the percentage of injected activity per gram of tissue (%IA/g). 

Epu 2 %ID/g=ATissueMTissueATotal*100

Where A_tissue_ is the ^68^Ga activity in the sample, M_tissue_ is the mass of the sample and A_total_ is the total activity of ^68^Ga injected into the rat (23, 24). 

*PET scan imaging*

The rats were anesthetized with ketamine 50 mg/kg and xylazine 5 mg/kg; the ^68^Ga-MAA was injected into the rats’ tail vein (25). Static PET/CT imaging of the first rat was performed 50 min after 6.66 MBq injection of ^68^Ga- MAA on the SIEMENS Biograph 6 clinical PET/CT scanner (26). The decay corrected acquisition protocol was set to 15 min per bed position. Figure 3 shows the distribution of ^68^Ga-MAA in the first rat via PET/CT and PET MIP images. 

**Table 1.** Decay corrected organ maximum activity concentration (Bq/mL) of ^68^Ga-MAA during the first 45 min

**Organ**	^68^ **Ga-** **MAA**								
	1 min	5 min	10 min	15 min	20 min	25 min	35 min	40 min	45 min	
Bladder	11990	45092	55371	61601	67875	54429	94294	81037	22838	
R. Kidney	32354	22703	15795	14808	13282	12653	11905	10603	9561	
L. Kidney	28374	20763	16618	14189	11945	12693	11362	12145	8703	
Lungs	145166	145371	147475	131815	142757	122164	110887	107585	97605	

**Figure 2:**
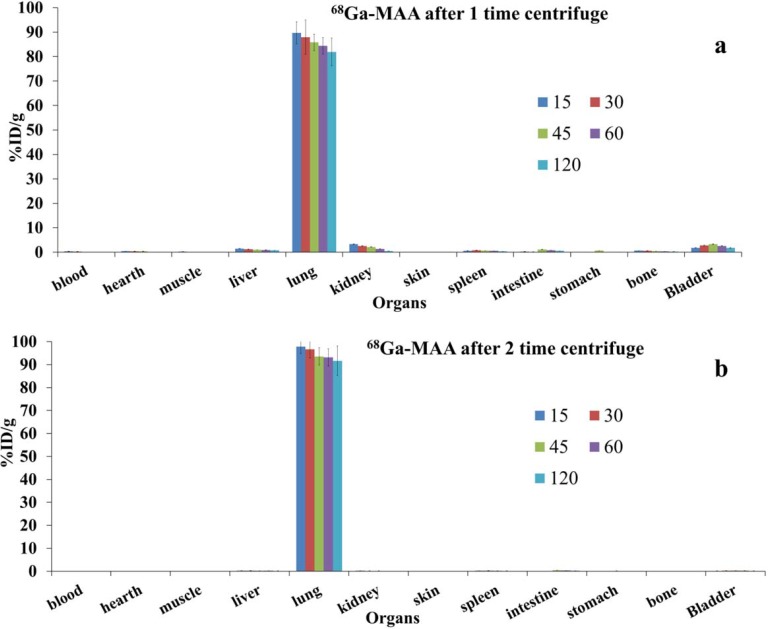
Biodistribution of ^68^Ga-MAA after 1 and 2 times centrifuge post labeling (the data were decay corrected) at various time points (from 15 to 120 min) post injection

**Figure 3:**
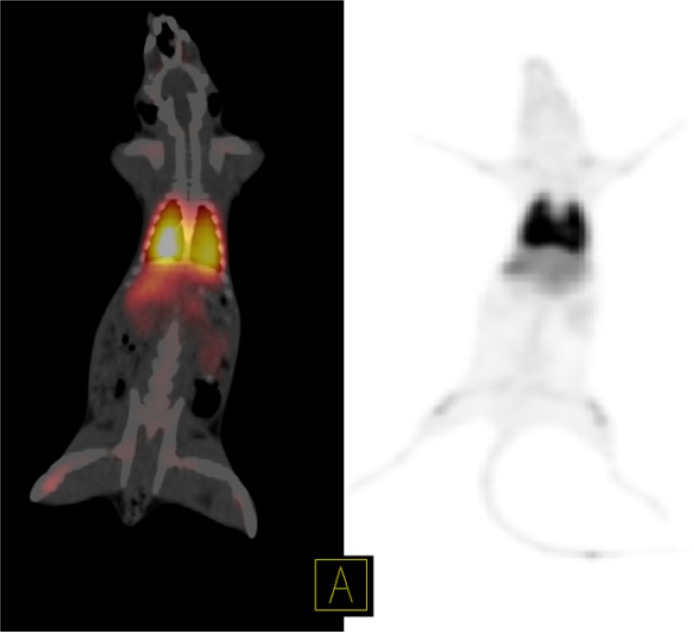
Static PET/CT fused image (left) and PET MIP image (right) of ^68^Ga-MAA in the first rat 50 min after injection show significant accumulation of ^68^Ga-MAA in lungs. The injected dose was 6.66 MBq

**Figure 4:**
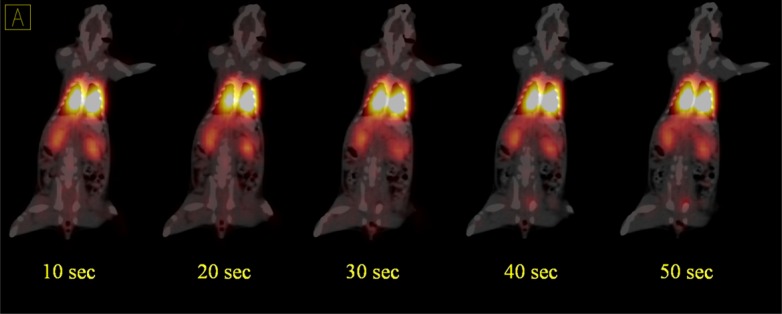
Dynamic PET/CT fused images of ^68^Ga-MAA in the second rat during the first minute after injection show quick absorption of ^68^Ga-MAA in lungs and both kidneys. The injected dose was 1.85 MBq

**Figure 5:**
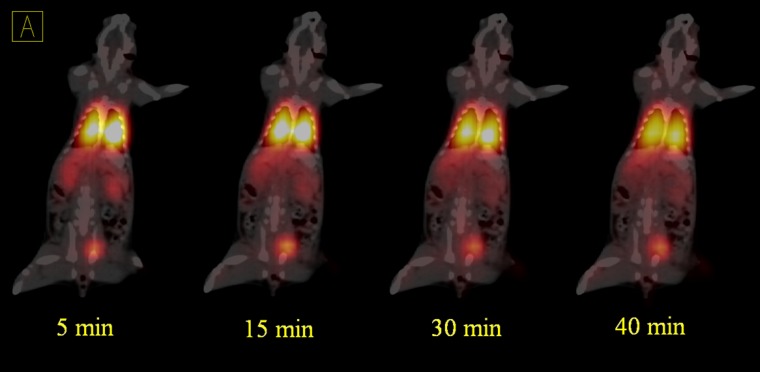
Dynamic PET/CT fused images of ^68^Ga-MAA in the second rat after 5, 15, 30 and 40 min after injection. The activity was extracted by the kidneys to the bladder

Dynamic PET/CT images were acquired for 45 min in list mode (32 bit) format a few sec after injection (Figures 4 and 5). In all of the acquisitions, the rats were placed in supine position and CT scans performed for anatomical reference and attenuation correction (spatial resolution 1.25 mm, 80 kV, 150 mAs) with a total CT scanning time of 20 sec. 

Reconstruction was performed using the TrueX algorithm (including resolution recovery) with attenuation correction. The reconstruction settings were 2 iterations and 21 subsets to a 336×336 matrix. Images were filtered with Gaussian filter of 5mm full-width half maximum (FWHM). Transmission data were reconstructed into a matrix of equal size by means of filtered back-projection, yielding a co-registered image set (26). 

## Results

The size distribution results were depicted in Figure 1. As shown in Figure 1, after centrifuges the size became more uniform and after two times the uniformity became better. The average sizes of the MAA particles were 24.31 µm, 25.79 and 26.44 before and after 1 time and 2 times centrifuge, respectively.

The biodistribution of ^68^Ga-MAA (with one and two times centrifuge post labeling) was represented in Figure 2. As shown in Figure 2 the lung took up the majority of the activities. Our results showed that centrifuging the final product, increased lung uptakes from 89% to 97% of the total injecting dose per gram (ID/g) after 5 min post injections.

^68^ *Ga-MAA Preclinical PET/CT Imaging*

The PET/CT of ^68^Ga-MAA after first centrifuge was represented in Figures 3, 4 and 5

## Discussion

Previous studies stated that size is an important issue on biodistribution of the particles (19, 25, 27, 28). If the particles are larger than 0.08 µm and less than 10 µm their target organs trough the i.v. injection were in the reticuloendothelial system (RES) and bones instead of Lungs (19, 27, 28). As represented in Figure 1, particle size measurements show that after first centrifuge about 1.31 % of MAA particles were less than 2 µm and about 5.2% were between 2-10 µm, which results the unwanted uptakes in liver and kidneys and about 0.6% undesirable bone marrow uptakes. The uninvited uptakes problem can be solved by centrifuge the ^68^Ga-MAA again; however, this purification procedure is takes 10 min more and losing the activities with the magnitude of 15-20%. As demonstrated in Figure 2 b after more purification the lung uptake will rise from 89% into 97% of %ID/g, which is in accordance with the previous studies on ^68^Ga-MAA (9-11), though none of the previous studies were not assess the uptakes of ^68^Ga-MAA before 1 h post injection. Therefore they could have some unwanted uptakes in liver or kidneys at 15 min post injection.

Although this study was in continues to the previous studies which discuss the advantages of the using ^68^Ga-MAA, but there is still a lack of data in terms of the kinetics of MAA in blood and lung also none of the previous studies discuss the undesirable uptakes in RES and in bone marrow. Therefore, the main advantages of this study are showing the uptakes and kinetics of ^68^Ga-MAA from the first minutes post injection and explain how to prevent these phenomena. As shown in Figure 3, the PET/CT and PET MIP (Maximum Intensity Projection) images of the first injected rat clearly show the significant activity concentration of ^68^Ga-MAA in lungs 50 min after injection. The low levels of uptake in other organs were clearly seen. During the first minute of ^68^Ga-MAA injection, as shown in Figure 4, quick uptake of lungs, and both kidneys are noticeable. Most of the injected activity was detected in the lungs and kidneys after tail vein injection (Figure 4) while after a few minutes, the main activity was extracted by the kidneys to the bladder as represented in Figure 5. For quantitative uptake behavior, maximum activity concentrations (Bq/mL) were measured for lungs, kidneys and bladder at different time points (as represented in Table 1). 

As shown in table 1, from bladder to kidney maximum activity concentration ratios were increased from nearly zero in the first minute to 5.7 and 6.25 for right and left kidneys in the 45 min time point, respectively. Furthermore, the lungs to right kidney and lungs to left kidney maximum activity concentration ratios were increased from 4.5 and 5.1 in the first minute to 10.2 and 11.2 in the 45 min post injection. While initial kidneys uptake was decreasing via excretion into the bladder, table 1 clearly shows the preferential allocation of ^68^Ga-MAA in the lungs over the period studied. 

There is another merit of ^68^Ga-MAA compares to the ^99m^Tc-MAA in terms of lung uptakes were other studies showed that the lung uptake for ^99m^Tc were about 87 and 79 after 2 and 4 h post injection, respectively this value for ^68^Ga-MAA is more than 90% (10, 11). This advantage let the physician prescribe the lower injected dose for ^68^Ga-MAA due to its higher uptakes and better physical characteristics of the PET compare to the SPECT (29).

## Conclusion

As RTLC showed, the centrifuges increase labeling yield from 90-95% for Pars-Isotopes and GE kits respectively to 100%. In addition, the microscopic size examination demonstrated a shift in the particles size post centrifuge. Moreover, the biodistribution data revealed the efficiency and benefits of the centrifuge in terms of preventing liver and bone marrow uptakes especially for Pars-Isotopes kits. In conclusion, the whole procedure took less than 25 min from elution to the ﬁnal product. Using ^68^Ga-MAA is recommended, because it remained shorter than ^99m^Tc-MAA in the lung and showed better image qualities.
